# Distribution of *Mycobacterium tuberculosis* Lineages and Drug Resistance in Upper Myanmar

**DOI:** 10.3390/tropicalmed7120448

**Published:** 2022-12-19

**Authors:** Aye Nyein Phyu, Si Thu Aung, Prasit Palittapongarnpim, Kyaw Ko Ko Htet, Surakameth Mahasirimongkol, Htin Lin Aung, Angkana Chaiprasert, Virasakdi Chongsuvivatwong

**Affiliations:** 1 National Tuberculosis Programme, Department of Public Health, Ministry of Health, Mandalay 05071, Myanmar; 2 Department of Epidemiology, Faculty of Medicine, Prince of Songkla University, Hat Yai 90110, Thailand; 3 Department of Public Health, Ministry of Health, Keng Tung 06231, Myanmar; 4 Pornchai Matangkasombut Center for Microbial Genomics, Department of Microbiology, Faculty of Science, Mahidol University, Bangkok 10400, Thailand; 5 Genomic Medicine and Innovation Support (GeMIS), Department of Medical Sciences, Ministry of Public Health, Bangkok 10400, Thailand; 6 Department of Microbiology and Immunology, University of Otago, Dunedin 9016, New Zealand; 7 Office of Research and Development, Faculty of Medicine Siriraj Hospital, Mahidol University, Bangkok 10400, Thailand

**Keywords:** *Mycobacterium tuberculosis* lineages, drug resistant TB, Upper Myanmar

## Abstract

*Mycobacterium tuberculosis* complex (MTBC) is divided into 9 whole genome sequencing (WGS) lineages. Among them, lineages 1–4 are widely distributed. Multi-drug resistant tuberculosis (MDR-TB) is a major public health threat. For effective TB control, there is a need to obtain genetic information on lineages of *Mycobacterium tuberculosis* (Mtb) and to understand distribution of lineages and drug resistance. This study aimed to describe the distribution of major lineages and drug resistance patterns of Mtb in Upper Myanmar. This was a cross-sectional study conducted with 506 sequenced isolates. We found that the most common lineage was lineage 2 (n = 223, 44.1%). The most common drug resistance mutation found was streptomycin (n = 44, 8.7%). Lineage 2 showed a higher number of MDR-TB compared to other lineages. There were significant associations between lineages of Mtb and drug resistance patterns, and between lineages and geographical locations of Upper Myanmar (*p* value < 0.001). This information on the distribution of Mtb lineages across the geographical areas will support a lot for the better understanding of TB transmission and control in Myanmar and other neighboring countries. Therefore, closer collaboration in cross border tuberculosis control is recommended.

## 1. Introduction

*Mycobacterium tuberculosis* complex (MTBC) is divided into nine lineages based on whole genome single nucleotide polymorphism. Among them, four major lineages are widely distributed all over the world [1].

Different parts of the world are dominated by different MTB lineages. This may lead to the hypothesis that the strain type is specifically slightly adapted to different human populations [2]. More than 80% of the global Lineage 1 burden was in India, the Philippines, Indonesia, and Bangladesh, which have some of the highest absolute numbers of TB cases in the world [3]. Lineage 2 migrates particularly from Asia to Europe and Africa and is associated with high incidence and transmission of multidrug-resistant tuberculosis (MDR-TB) [4]. Lineage 3 strains represent the major tuberculosis (TB) burden in high-incidence regions of South Asia, North Africa, and East Africa, and are also potential drivers of MDR-TB epidemics in some parts of the world [5]. Lineage 4 is common in Europe and America in areas with high TB rates and high Human Immunodeficiency Virus (HIV) co-infection [6].

MDR-TB is a major global health threat. In 2021, drug-resistant TB (DR-TB) is estimated to have increased between 2020 and 2021, with 450,000 (95% Uncertainty Interval (UI): 399,000–501,000) new cases of rifampicin-resistant TB (RR-TB) annually. In Myanmar, the incidence of MDR/RR-TB was 18 (range 10–26) per 100,000 population in 2021 [7]. Laboratory confirmed cases of MDR-TB were 2368 in 2020 [8].

For effective TB control and prevention, it is necessary to understand lineages and drug resistance in the specific country. There was only scant information about this relationship in low-income countries such as Myanmar. This was the first study in Upper Myanmar and it improved the new knowledge of genomic variants associated with drug resistance in Mtb. Moreover, nowadays, individualized treatments are needed depending on the drug resistant patterns of each TB patient in order to increase treatment success rates, reduce treatment side-effects and complications, and to get better treatment outcomes. In addition, understanding geographical distribution of the lineages and their relationship with drug resistance could help health policy makers to plan for proper intervention programs. The objectives of this study were to describe the geographical distribution of major lineages and drug resistance patterns of Mtb in regions and states of Upper Myanmar.

## 2. Materials and Methods

### 2.1. Study Sites

**Figure 1:**
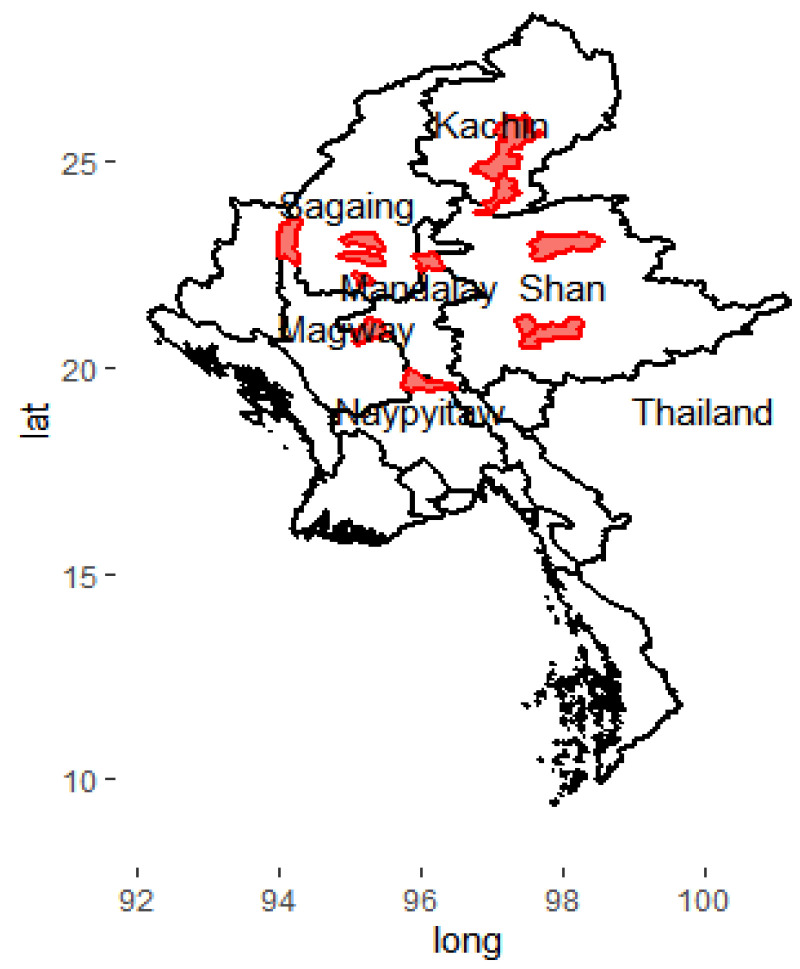
Selected townships among regions and states of Upper Myanmar. The red colour indicates the selected study sites.

Myanmar is divided into seven regions, seven states, and one Union Territory. The population of Myanmar was 54 million in 2021. The total TB incidence was 194,000 (134,000–264,000) in 2021 [7]. Upper Myanmar is in the central, northernmost, and easternmost parts of the country. Upper Myanmar consists of Sagaing, Mandalay, Nay Pyi Taw, Magway regions, and Kachin and Shan states. This study was carried out at TB clinics of Public Health offices in 18 selected townships among regions and states of Upper Myanmar (Figure 1).

### 2.2. Study Design

Thus, a cross-sectional study was conducted between February and August 2020. Study participants were bacteriologically confirmed TB patients based on sputum smear microscopy for acid fast bacilli and/or Gene Xpert MTB/RIF Assay. The exclusion criteria were patients with extra-pulmonary TB, sputum smear negative patients that were also “MTB not detected” by X-pert MTB/RIF test, patients that had received more than 7 days of Anti-TB treatment in their current treatment course, and prisoners. Among townships with more than 10 of bacteriologically confirmed TB cases in Upper Myanmar, 18 eligible sites were selected in the survey.

### 2.3. Participants

To draw a representative sample of townships where the study TB clinics were located, a sampling frame was assembled which comprised the list of 167 eligible townships in the country. The systematic random sampling method was used to select the required number of TB patients from all eligible townships according to the following procedures. First, to select the TB clinics, a cumulative case count of the sampling frame was compiled. The TB clinic was the primary sampling unit for sampling. Second, the total number of notified smear positive TB patients in each TB clinic was divided by 50 to obtain the sampling interval (i). Third, a random number between 1 and the sampling interval (i) was then picked (x). Finally, using the cumulative case count, the townships corresponding to a multiple of xth patients were selected as the study clinic [9]. For Upper Myanmar, 18 study TB clinics were included. In each clinic, 40 consecutive eligible TB patients were recruited.

**Figure 2:**
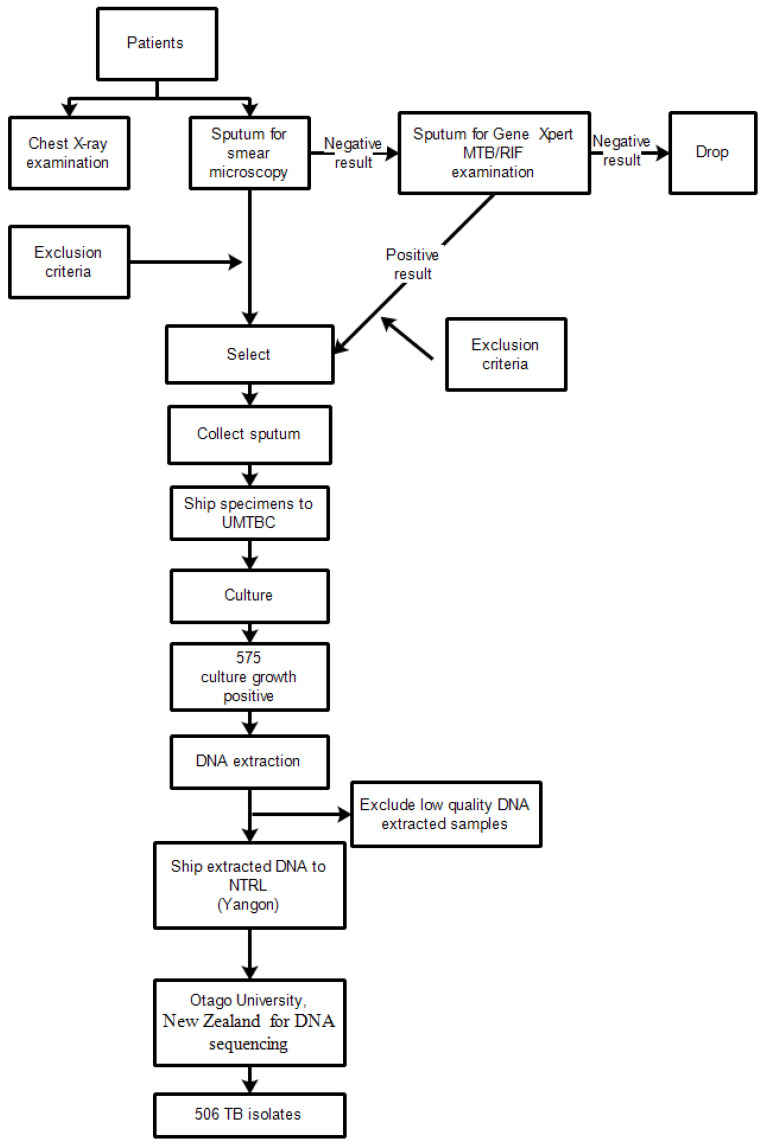
Laboratory workflow at the culture facility in Upper Myanmar and Otago University, New Zealand.

Firstly, eligible TB patients with positive sputum smear were identified. After that, informed consent or assent was taken from the participants with the informed consent form. Then, the TB coordinator proceeded to interview the patient by using pre-tested, structured questionnaires and cross-check the medical records to allow classification of patients into new or previously treated. The enrolled patient was assigned a survey ID which was then noted in the Township TB Laboratory Register. The laboratory technician, in coordination with the TB coordinator, arranged a shipment of survey specimens and forms to the Upper Myanmar TB Center and Laboratory as quickly as possible. A flow chart of the laboratory algorithm for the survey is displayed in Figure 2. For sputum smear positive TB patients, three early morning sputum samples (one for Gene Xpert and two for culture) (3–5 mL/sample, minimum 3 mL) were directly sent to the Upper Myanmar TB Laboratory without testing by Xpert MTB/RIF in township/district TB laboratories.

If the smear was negative with any CXR abnormalities, three early morning sputum samples would be asked from the patient: one for Gene Xpert and two for culture. Gene Xpert MTB/RIF testing was performed in TB laboratories that had the Gene Xpert machine available, either in the district or the Upper Myanmar TB Laboratory. If the Xpert MTB/RIF result was negative, the other two samples would be discarded and this patient would not be included in the survey. If the Xpert MTB/RIF result was positive, the remaining two samples were sent to the Upper Myanmar TB Laboratory for culture by MGIT immediately on arrival.

### 2.4. Laboratory Processing of Specimens

A total of 637 TB patients was included in this study. Among the 637 smear positive or Xpert MTB/RIF positive samples, there were 575 positive cultures and Deoxyribonucleic acid (DNA) extraction was done. After excluding low quality DNA extracted samples, WGS of 506 isolates were available for further analysis.

### 2.5. Extraction of Genomic DNA, Whole Genome Sequencing, Variant Calling, Classification of Lineages and Prediction of Drug Resistance

At the Upper Myanmar TB Laboratory with biosafety level 3, genomic DNA was extracted from cultures of a single sputum specimen of each patient by using MoBio Microbial DNA Isolation Kits (https://www.qiagen.com, accessed on 14 May 2020) and sequenced by using Illumina MiSeq (https://www.illumina.com, accessed on 5 February 2021) at Otago University, New Zealand as previously described [10,11]. Both first- and second-line LPA (Line Probe Assay) were tested by the MTB DRplus kit and MTB DRsl kit.

The sequencing library was prepared using the NexteraTM XT DNA Kit and subjected to LabChip^®^ DNA High Sensitivity Assay on the LabChip GX Touch HT platform (Perkin Elmer, Waltham, MA, USA) for the quality control check. The resulting library was then subjected to sequencing using paired-end 250-bp reads on an Illumina MiSeq according to the manufacturer’s instructions (Illumina Inc., Hayward, CA, USA).

The resulting sequencing reads were mapped to the H37Rv reference genome (Gene Bank accession no. NC_000962.3) using the in-house pipeline based on a combination of bcf and sam tools. PhyResSE and TB Profiler were used for strain/lineage classification. The drug resistant genes that are commonly used to treat drug-susceptible and MDR-TB cases in Upper Myanmar were identified. Online analysis pipelines PhyResSE and TB Profiler were also used to confirm and crosscheck all WGS results.

### 2.6. Statistical Analysis

Data entry was done using EpiData version 3.1 (EpiData Association, Odense, Denmark) and data were analysed using R version 4.0.2 (R foundation for Statistical Computing, Vienna, Austria). The background characteristics of TB patients, Mtb lineages, and their drug resistance mutations and patterns were presented as frequency and percentage. The relationship between lineages and drug resistance patterns, as well as the geographical distribution of lineages and drug resistance patterns according to regions and states of Upper Myanmar, were analyzed using Fisher’s exact test. The significant level was set at *p* value less than 0.05.

## 3. Results

### 3.1. Background Characteristics of Participants

Among 506 TB patients, 22.3% were aged 31–40 years, 68.4% were male, 11.7% had diabetes mellitus, 22.7% were smokers, and 7.9% had a previous history of TB treatment in Table 1.

### 3.2. Distribution of Lineages

Four lineages were identified among 506 TB patients via WGS in Table 2. Among them, lineage 2 accounted for 44.1% of the isolates, followed by lineage 1 (39.7%), lineage 4 (12.3%), and lineage 3 (4.0%), respectively.

### 3.3. Type of Anti-TB Drug Resistance by WGS

Among 506 isolates, 88.1% were sensitive to all anti-TB drugs in Table 3. 5.1% were mono resistant to streptomycin. 1.6% were mono resistant to isoniazid. 0.4% were mono resistant to levofloxacin. 4.7% were poly resistant. 1.8% were multi-drug resistant. 1.0% were Pre-Extensively Drug Resistant Tuberculosis (Pre-XDR-TB, defined as TB that is resistant to rifampicin and any fluoroquinolone) [7].

### 3.4. Association between Lineages and Drug Resistance Mutations

Significant association was found between lineages and drug resistance mutations (*p* value = 0.017) in Table 4. Moreover, lineage 2 shows the higher number of drug resistance mutations compared to other lineages.

### 3.5. Association between Lineages and Drug Resistance Patterns

Table 5 shows the association between lineages and drug resistance patterns. There were 7 Anti-TB drug resistances predicted from WGS. This can result in 128 possible combinations (2^7^ = 128). Out of these, only 14 patterns were found in the data. We display the six most common patterns and combine the remaining into two rows and tested the association with lineage using Fisher’s exact test. The result gives a very significant *p* value of <0.001. There was a distinct linkage between lineage 2 and the common drug resistant mutations related to streptomycin, isoniazid, and also rifampicin. Moreover, lineage 2 shows the higher number of MDR compared to other lineages.

### 3.6. Geographical Distribution of Lineages and Drug Resistance Patterns

Table 6 shows the geographical distribution of lineages and drug resistance patterns according to the States and Regions of Upper Myanmar. There were significant associations between the lineages and geographical locations of Upper Myanmar (*p* value < 0.001). Shan and Kachin had a higher percentage of lineage 2 compared to other regions. There was no significant association between drug resistance patterns and geographical locations.

## 4. Discussion

This study included the 506 TB patients who were visited at the TB clinics in 18 selected townships among the regions and states of Upper Myanmar during the data collection period of 1 February 2020 to 31 August 2020. Among them, nearly one fourth of them were 31–40 years, about two thirds were male, and about the same proportion did not have diabetes. Over half of them were non-smokers and the majority of the patients had no history of TB treatment. In this study, four types of Mtb lineages were identified via WGS and we found that lineage 2 was the most common lineage, followed by lineage 1, lineage 4, and lineage 3, respectively.

Many studies have shown the distribution of Mtb lineages in Southeast Asia. A previous study by Moe Sann et al. in Northern Myanmar revealed that lineage 2 was predominant (89.23%). The others were distributed among lineage 1 (3.08%), lineage 3 (1.54%), and unknown (6.15%). Lineage distributions are not uniform and the predominance of lineage 2 may be due to the human interaction with foreign countries such as China, India, and Bangladesh [12]. It is not clear where the origins of lineage 4 in Upper Myanmar are located. Although lineage 4 is also called the Euro-American strain, most of the lineage 4 in Chiangrai province, Thailand were L4.4 and L4.5, which may be originally from China [13]. Although Myanmar has a long history of relations with Europeans, the lineage distribution in Upper Myanmar was rather different from Europe or America. For example, a study in Florida from 2009 to 2015 revealed that the majority (83.4%) of the cases were infected with lineage 4 [14]. In Europe and South Africa, lineage 4 is common in areas with high levels of tuberculosis [6].

Table 7 compares the distribution of Mtb lineages from our study with those of other studies [13,15,16,17,18,19].

The distribution of the lineages in our study was most similar to that in Thailand, where lineages 2 and 1 are in close and higher proportion. Interestingly, our pattern was quite different from those in other sites in Myanmar—such as Yangon, where only lineage 2 was predominant, and in Kayin, where only lineage 1 predominated.

Regarding the type of anti-TB drug resistance, we found poly drug resistance, MDR-TB, and pre-XDR TB, but no Extensively Drug Resistant TB (XDR-TB, resistance to rifampicin, any fluoroquinolone and at least one of bedaquiline or linezolid) [7]. We also found mono drug resistant to streptomycin, levofloxacin, and isoniazid. In Germany, mono drug resistant to streptomycin was both the most prevalent form of drug resistance and the most frequent drug resistance among multidrug-resistant (MDR) strains [20]. In our study, resistance to new and repurposed drugs such as bedaquiline, delamanid, linezolid, and clofazimine was not found. This information will be very useful for National TB Programme, Myanmar, which is starting to use new oral regimens for MDR-TB patients [21].

Concerning the distribution of drug resistant mutations, there were 32 resistant streptomycin isolates with a *rpsL* K43R mutation, which was the most common occurrence in Upper Myanmar, followed by 22 resistant isoniazid isolates with *katG* S315T mutation. Streptomycin resistance was the first Mtb drug resistance described [22]. A whole genome-based study suggested that resistance mutations to isoniazid and streptomycin, the first two anti-TB drugs used in history, were precursors of MDR TB strains [23]. Streptomycin drug resistance was also significantly associated with lineage 2 of Mtb in the Myanmar-Thailand Border Area [16]. In our study, there were significant associations between lineages and drug resistance mutations. Moreover, the type and frequency of streptomycin resistance-conferring mutations have been shown to vary according to the geographical region [24,25,26,27,28,29,30,31,32,33,34,35,36]. For instance, mutations in *rpsL* were relatively higher in Asia [26,30,33,34]. In addition, the K43R mutation was associated with the MTB Beijing genotype [30,33,34]. Many studies report that mutations in *rpsL*, *rrs*, and *gidB* are responsible for resistance to streptomycin, the drug that is commonly used in both multidrug-resistant tuberculosis (MDR-TB) treatment and retreatment in Myanmar [37]. In addition, streptomycin is also involved in constructing special regimens for hepato-toxic patients who are not appropriate for routine anti-TB treatment regimen.

Significant association was found between lineages and drug resistance patterns in our study. Previous studies have not only shown that the tuberculosis epidemic in Eastern Europe is more complex and driven by lineage 2, but also documented an association between lineage 2 and MDR-TB [4]. A WGS study from Yangon, Myanmar, identified a strong association between lineage 2 and drug resistance [22]. There was also a significant association between Lineage 2 and MDR-TB in our study, which was similar to previous studies.

In this study, there were significant associations between lineages and geographical locations of Upper Myanmar. Several studies have described the distribution of Mtb lineage and its association with the geographical background of tuberculosis patients in other parts of Southeast Asia [13,17,38,39]. Moreover, Upper Myanmar has closer lineage patterns to Thailand, suggesting possible cross border transmission in the two areas. This information on the distribution of the Mtb lineage can be linked in the future to that on the other side of the border to assess cross-border transmission [15,39,40].

Our study has major implications. Results from this study could be used to develop more effective DR-TB guidelines and to construct the drug regimens that might be most clinically potent for a particular patient. Findings from this study are very important for country disease burden re-estimation, National Strategic Plan resetting, upcoming national forecasting, and quantification of National TB Programme.

Our study carries some limitations. The study was only representative for new smear-positive TB patients as the sample size determination was designed for this category of patients. Therefore, the current findings are not fully representative for smear-negative and extra-pulmonary TB patients.

## 5. Conclusions

Our study revealed the distribution of Mtb lineages across the geographical areas in order to increase our knowledge of genomic diversity associated with drug resistance in Mtb, as well as to better understand the TB transmission and control in Myanmar. Moreover, resistance to streptomycin and isoniazid had higher prevalence. Their role in anti-TB drug is becoming limitted and should be reviewed regularly.

## Figures and Tables

**Table 1:** Background characteristics of participants.

Variables	N = 506 (%)
Age in year	
<20	40 (7.9)
20–30	93 (18.4)
31–40	113 (22.3)
41–50	106 (20.9)
51–60	90 (17.8)
>60	64 (12.6)
Gender	
Male	346 (68.4)
Female	160 (31.6)
Diabetes mellitus status	
Positive	59 (11.7)
Negative	348 (68.8)
Unknown	99 (19.6)
Smoking status	
Yes	115 (22.7)
Never	268 (53.0)
Ex-smoker	123 (24.3)
Previously treated for TB	
Yes	40 (7.9)
No	465 (91.9)
Unknown	1 (0.2)

**Table 2:** Distribution of lineages.

Lineages	N = 506 (%)
Lineage 1	201 (39.7)
Lineage 2	223 (44.1)
Lineage 3	20 (4.0)
Lineage 4	62 (12.3)

**Table 3:** Type of Anti-TB Drug Resistance.

Type of Anti-TB Drug Resistance	N (%)
Sensitive to all drugs	446 (88.1)
Any drug resistance	60 (11.9)
Mono drug resistance to streptomycin	26 (5.1)
Mono drug resistance to isoniazid	8 (1.6)
Mono drug resistance to levofloxacin	2 (0.4)
Poly drug resistance	24 (4.7)
Multi-drug resistance	9 (1.8)
Pre-XDR-TB	5 (1.0)

**Table 4:** Association between lineages and drug resistance mutations.

Drug Resistance Mutation	Total	Lineages				Fisher’s Exact Test, *p* Value
		1	2	3	4	
	N	(N = 201)	(N = 223)	(N = 20)	(N = 62)	
Drug resistant mutation						0.017
Isoniazid						
*katG*_S315T	22	3	14	1	4	
*InhA*_C15T	9	2	4	1	2	
*oxyR-ahpC*_G-48A ap	1	0	1	0	0	
Rifampicin						
*rpoB*_S450L	7	1	5	0	1	
*rpoB*_I491F	1	0	1	0	0	
*rpoB*_H445D	1	0	1	0	0	
Pyrazinamide (Z)						
*ncA*_408_ins_1_a_at	1	0	1	0	0	
Ethambutol (E)						
*embB*_M306V	3	0	2	0	1	
*embB*_M306I	1	0	1	0	0	
*embC*_c-516t	1	0	1	0	0	
Streptomycin (S)						
*rpsL*_K43R	32	2	28	1	1	
*rpsL*_K88R	7	1	6	0	0	
*gidB*_A138V	3	2	0	0	1	
*rrs*_C517T	1	1	0	0	0	
*gidB*_L91P_gid	1	0	1	0	0	
Levofloxacin (Lfx)						
*gyrA*_D94G	3	0	2	0	1	
*gyrA*_D94N	1	0	1	0	0	
*gyrA*_A90V	3	1	1	0	1	
Ethionamide (Eto)						
*inhA*_C15T	6	1	3	1	1	

**Table 5:** Association between lineages and drug resistance pattern.

Drug Resistance Pattern	Lineages				Fisher’s Exact Test, *p* Value
	1	2	3	4	
	(N = 201)	(N = 223)	(N = 20)	(N = 62)	
Drug resistance patterns					<0.001
H_0_ R_0_ Z_0_ E_0_ S_0_ Lfx_0_ Eto_0_	193	181	18	54	
H_0_ R_0_ Z_0_ E_0_ S_R_ Lfx_0_ Eto_0_	3	22	0	1	
H_R_ R_0_ Z_0_ E_0_ S_0_ Lfx_0_ Eto_0_	2	2	0	4	
H_R_ R_0_ Z_0_ E_0_ S_R_ Lfx_0_ Eto_0_	1	6	1	0	
H_R_ R_0_ Z_0_ E_0_ S_0_ Lfx_0_ Eto_R_	0	2	1	1	
H_R_ R_R_ Z_0_ E_0_ S_R_ Lfx_0_ Eto_0_	0	3	0	0	
Others including H_R_ R_R_	1	4	0	1	
Others not including H_R_ R_R_	1	3	0	1	

Subscript 0 denotes sensitive. Subscript R denotes resistant. E = Ethambutol; Eto = Ethionamide; H = Isoniazid; Lfx = Levofloxacin; R = Rifampicin; S = Streptomycin; Z = Pyrazinamide.

**Table 6:** Geographical distribution of lineages and drug resistance pattern.

Lineages and Drug Resistance Patterns	Regions and States of Upper Myanmar						Fisher’s Exact Test, *p* Value
	Regions				States		
	Sagaing (N = 105)	Magway (N = 72)	Naypyitaw (N = 34)	Mandalay (N = 174)	Shan (N = 56)	Kachin (N = 65)	
**(A) Lineages**							<0.001
Lineage 1	51	33	15	79	6	17	
Lineage 2	39	28	13	63	40	40	
Lineage 3	4	3	0	10	1	2	
Lineage 4	11	8	6	22	9	6	
**(B) Drug Resistance Patterns**							0.123
H_0_ R_0_ Z_0_ E_0_ S_0_Lfx_0_ Eto_0_	91	66	31	158	43	57	
H_0_ R_0_ Z_0_ E_0_ S_R_ Lfx_0_ Eto_0_	4	3	2	5	8	4	
H_R_ R_0_ Z_0_ E_0_ S_0_ Lfx_0_ Eto_0_	3	2	1	2	0	0	
H_R_ R_0_ Z_0_ E_0_ S_R_ Lfx_0_ Eto_0_	2	0	0	3	1	2	
H_R_ R_0_ Z_0_ E_0_ S_0_ Lfx_0_ Eto_R_	1	0	0	1	2	0	
H_R_ R_R_ Z_0_ E_0_ S_R_ Lfx_0_ Eto_0_	2	0	0	0	1	0	
**Others**	2	1	0	5	1	2	

**Table 7:** N (%) of Mtb lineages according to our study and other studies.

	Our Study	Yangon, Myanmar	Kayin, Myanmar	Thailand	Philippines	Nepal	Tigray, Ethiopia
Lineage 1	201 (39.7)	9 (13)	73 (67)	480 (41)	143 (80)	32 (6)	1 (1.5)
Lineage 2	223 (44.1)	55 (76)	26 (23)	521 (45)	2 (1)	241 (48)	1 (1.5)
Lineage 3	20 (4.0)	4 (5)	4 (4)	11 (1)	0 (0)	153 (32)	28 (41.2)
Lineage 4	62 (12.3)	4 (5)	6 (6)	158 (13)	33 (19)	72 (14)	38 (55.8)
**Total**	506 (100.0)	72 (100)	109 (100)	1170 (100)	178 (100)	498 (100)	68 (100.0)

## Data Availability

All data analyzed are included in this published article.
